# Investigation of the interaction conditions of As_2_S_5_ and CuCl_2_ compounds in water and ethylene glycol medium

**DOI:** 10.55730/1300-0527.3722

**Published:** 2025-01-21

**Authors:** Huseyn IMANOV, Mahnur JAFARLI

**Affiliations:** Department of Chemistry, Faculty of Natural Sciences and Agriculture, Nakhchivan State University, Nakhchivan, Azerbaijan

**Keywords:** Differential thermogravimetric analysis, solution, micromorphology, element analysis, sedimentation, X-ray diffraction

## Abstract

The article presents the results of the interaction conditions of CuCl_2_ and As_2_S_5_ in ethylene glycol and aquatic medium. Besides, the results of the obtained samples’ physicochemical properties are given by X-ray diffraction (XRD), differential thermogravimetric (DTG), thermogravimetric (TG), elemental, and scanning electron microscope (SEM) methods. It was found that when ethylene glycol and water are used as a solvent and CuCl_2_ and As_2_S_5_ as the initial components, Cu_3_AsS_4_ is obtained at a temperature of 323–353 K. According to the results of DTA analysis, it was found that the melting point of obtained samples was 964.4 K contrary to the reactions carried out to obtain Cu_3_(AsS_4_)_2_ compounds both in ethylene glycol and water medium. Elemental analysis of the compounds was carried out, energy-dispersion spectrum, mass, and atomic ratios of copper, arsenic, and sulfur were determined, and the stoichiometric composition of Cu_3_AsS_4_ was determined. Furthermore, X-ray analysis of the obtained samples confirmed that peaks on the graphs correspond to the standard intensity maxima of the Cu_3_AsS_4_ compound.

## 1. Introduction

The unique structures, new chemical and physical properties of ternary chalcogenides are of great importance for application in nano-sized devices. These compounds have been extensively studied by materials science and nanotechnology researchers [1]. When a third element is added, these ternary chalcogenides are expected to be more significant due to the stoichiometric shift and synergistic effect than binary compounds [2]. They are used in transverse energy conversion (photovoltaic, photocatalysis, thermoelectricity), energy storage (lithium-ion batteries, hydrogen production), emission materials (plasmonics, LED, biological labeling), sensors (electrochemical, biochemical), biomedical devices (magnetic resonance imaging, X-ray computed tomography) and medical therapy (photochemotherapy treatments, immunotherapy, radiotherapy and drug delivery).

In [3], the semiconductor properties of natural samples of enargite (Cu_3_AsS_4_) containing copper and arsenic sulfide were studied. It was found to be p-type semiconductors with a resistivity of about 7 Ω cm, a doping level of about 1017 cm^−3^, and a charge of 9 cm^2^ V^−1^ s^−1^. The authors of [4] studied electrochemical oxidation and reduction of natural enargite (Cu_3_AsS_4_) surface in 0.1 M HCl solution using cyclic voltammetry and chronoamperometry. It was emphasized that Cu_6_As_4_S_9_ (sinnerite) and Cu_4_As_2_S_5_ (monoclinic: a = 1035.0 pm; b = 1465.0 pm; c = 3334.0 pm; β = 96) are decomposed by peritectic reaction. In other study, Cu-based systems were studied relatively intensively^1^. Sulfosalts of these systems include tetrahedrite (Cu_12+x_Sb_4+y_S_13_, where 0 ≤ x ≤ 1.92 and −0.02 ≤ y ≤ 0.27) and tennantite (Cu_12+x_As_4+y_S_13_, where 0 < x < 1.72 and 0 < 0.08) and some reported to be the most stable compared to other sulfide phases. In [5], three phases in the Cu-As-S system-luzonite, enargite, tennantite, lautite (CuAsS), sinnerite (Cu_6_As_4_S_9_) and new compound Cu_24_As_12_S_31_ were investigated. The only compound with a complex composition is tennantite, for which the generalized formula Cu_12+x_As_4+y_S_13_ was proposed, where 0 ≤ x ≤ 1.72 and 0 ≤ y ≤ 0.08. A number of ternary compounds found in nature were synthesized by the authors of [6]: enargite Cu_3_AsS_4_; famatinite Cu_3_SbS_4_; tetrahedrite Cu_3_SbS_3_; tennantite Cu_3_AsS_3_; wolfsbergite CuSbS_2_; and proustite Ag_3_AsS_3_. Several new compounds corresponding to the stoichiometry of these natural compounds have also been synthesized: Cu_3_SbSe_4_, CuSbSe_2_, CuAsS_2_, CuAsSe_2_, AgSbSe_2_, AgSbTe_2_, and Ag_3_AsSe_3_. All compounds are semiconductors.

Copper-arsenic-sulfide (Cu_3_AsS_4_) thin films were prepared for the first time by deposition of nanoparticles in an arsenic(V) sulfide atmosphere [7]. When the resulting thin layers are thermally treated at 425 0C, the crystal structure changes from tetragonal to orthorhombic. In the other study, Cu-As-S thin layers were deposited using the thermal evaporation method^2^. Optical emission spectra of Cu_0.1_As_2.1_S_3.1_ thin films were measured in the temperature range of 77–300 K. Closely spaced micron-sized copper-arsenic-sulfide (Cu_3_AsS_4_) thin films were synthesized by heating nanoparticles in As_2_S_5_ [8]. RFA revealed the transformation of tetragonal Cu_3_AsS_4_ nanoparticles into an orthorhombic structure, and Raman spectroscopy revealed the presence of a second amorphous phase. Enargite Cu_3_AsS_4_ is a promising absorber for thin-film solar cells [9–11]. However, there is little information about optoelectric properties [12]. The authors suggested adding other buffer layers than CdS to obtain solar cells from Cu_3_AsS_4_.

In [13], the thermal decomposition of enargite (Cu_3_AsS_4_) in a nitrogen environment was investigated by TG analysis in the temperature range of 575–700 0C. It was found that the decomposition proceeds in two successive stages with the formation of tenantite (Cu_12_As_4_S_13_) as an intermediate compound. In [14], the deposition of enargite (Cu_3_AsS_4_) in the temperature range of 648 K–898 K in an atmosphere with a variable amount of oxygen was studied by the TG analysis method. Based on the mass loss and gain data and RFA results, the mechanism of enargite oxidation reaction, which occurs in three successive stages. In [15], the reaction paths in the synthesis of Cu_3_AsS_4_ were studied by the DTA method in the range of 25–870 0C, with a heating rate of 10 K/min. Various intermediates were found to exist mainly as binary compounds (CuS and As_4_S_4_) at low temperatures and ternary compounds containing Cu_6_As_4_S_9_, Cu_4_As_2_S_5_ or Cu_12+x_As_4+y_S_13_ at higher temperatures. It was noted that heating Cu_3_AsS_4_ above its melting point at 694 0C increases the amount of byproducts. The structure of synthetic tennantite-Cu_12_As_4_S_13_ was studied at different temperatures in the range of 90–293 K [16].

In [17] and [18], phase equilibria respectively in the Cu_2_S–Cu_3_AsS_4_–S and Cu-As-S systems were studied by DTA and RFA. In the Cu_2_S–Cu_3_AsS_4_–S system, important graphs characterizing, namely T–x diagrams of Cu_2_S–Cu_3_AsS_4_ and Cu_3_AsS_4_–S quasibinary systems, isothermal section of the phase diagram at 300 K, and the projection of the liquidus surface have been constructed. In the Cu-As-S system, the fields of primary crystallization of phases, types, and coordinates of invariant and monovariant phase equilibria are determined. In [18], the presented phase diagram reflects four ternary compounds Cu_3_AsS_4_, Cu_12_As_4_S_13_, Cu_6_As_4_S_9_, and CuAsS, which are synthetic analogues of natural copper-arsenic sulfide minerals. The phase diagram of Cu_2_S-As, As-Cu_3_AsS_3_, Cu_3_AsS-Cu_2_S and Cu_2_S-As_2_S_3_ quasibinary cross sections is constructed [19]. The last of these covers most of the ternary compounds. It is noted that the previously reported CuAsS_2_ compound was not observed.

It is clear from the above literature that very little is known about the interaction conditions of CuCl_2_ and As_2_S_5_ compounds in the solutions of organic and aquatic mediums. Taking this into consideration, we aimed to investigate the interaction between CuCl_2_ and As_2_S_5_ in an ethylene glycol and aquatic medium and present the results of the study.

## 2. Materials and methods

Arsenic(V) sulfide which was precipitated in ethylene glycol medium for this research was initially obtained using chemicals sodium arsenate, hydrochloric acid, and hydrogen sulfide gas. Furthermore, arsenic(V) sulfide which is obtained in high purity, and copper (II) chloride were used to obtain the copperthioarsenate compound. All chemicals used in experimental studies were provided as high-purity products and whole reactions were conducted in ethylene glycol and water medium. Besides, during the reactions, ammonia and nitrate solutions were used to maintain the pH of the medium at the desired level and distilled water and ethanol were used to wash the obtained precipitate. Moreover, ammonium molybdate and hydrazine solutions were used to analyze arsenic in sediment samples and leachate. All of the chemicals used during this study are of analytical grade.

### 2.1. Instrumental techniques

The following instruments were used for measurement and characterization: X-ray diffraction (XRD) analysis of samples was performed by a Bruker D2 Phaser XRD. Thermogravimetric (TG) and differential thermal analysis (DTA) data were recorded using the NETZSCH STA 449F3 instrument. A scanning electron microscopy (SEM) image for the investigation of the surface morphology is taken by the HITACHI TM3000 microscope. By the JSM-6610LV-SEM, elemental analysis of the composition of the obtained compound was carried out, and electronic images were taken to determine the stoichiometric composition of the compound. Glassco 710.AG.01 magnetic heater & stirrer (350 0C/1600 RPM) have been used for heating and stirring reaction solution. The pH measurements were carried out with an “аквилон” pH–410 at room temperature. “КФК – 2 – УХЛ 4.2” photo calorimeter was used to determine the arsenic content of the initial reaction filtrate and the precipitate obtained. The Berghof Speedwave professional chemical microwave oven has been used for the formation of sediment. A ВТ 20-21 liquid thermostat was used to conduct the reactions at stable temperatures.

### 2.2. Experimental part

In order to study the nature of physicochemical interaction between arsenic(V) sulfide and copper(II) chloride compounds in ethylene glycol and water environments, As_2_S_5_ compound was initially synthesized separately in ethylene glycol and water environments, respectively. For this, a certain amount of sodium arsenate was dissolved separately in ethylene glycol and water, the environment of both solutions was acidified with 10 N hydrochloric acid (pH = 0–1), and arsenic(V) sulfide was produced by releasing hydrogen sulfide gas at a temperature range of 273–283 K for 2 h. To carry out the reaction at a low temperature, the reaction mixture was placed in a ВТ 20-21 liquid thermostat. At this time, the following reaction occurs:


(1) $$2{\text{Na}}_{3}{\text{AsO}}_{4}+5{\text{H}}_{2}\text{S}+6\text{HCl}\overset{{\text{H}}_{2}\text{O}/{\text{C}}_{2}{\text{H}}_{6}{\text{O}}_{2}}{\to }{\text{As}}_{2}{\text{S}}_{5}+6\text{NaCl}+8{\text{H}}_{2}\text{O}$$

It is intended to obtain copper(II) thioarsenate compound from the interaction of arsenic(V) sulfide and copper(II) chloride in the environment of water and ethylene glycol according to the following reaction equations:


(2) $$8{\text{As}}_{2}{\text{S}}_{5}+15{\text{CuCl}}_{2}+24{\text{H}}_{2}\text{O}\to 5{\text{Cu}}_{3}{\left({\text{AsS}}_{4}\right)}_{2}+6{\text{H}}_{3}{\text{AsO}}_{4}+15\text{HCl}$$

(3) $${\text{CuCl}}_{2}.2{\text{H}}_{2}\text{O}+{\text{C}}_{2}{\text{H}}_{6}{\text{O}}_{2}\to \text{Cu}({\text{C}}_{2}{\text{H}}_{4}{\text{O}}_{2})+2\text{HCl}+2{\text{H}}_{2}\text{O}$$

(4) $$5\text{Cu}({\text{C}}_{2}{\text{H}}_{4}{\text{O}}_{2})+2{\text{As}}_{2}{\text{S}}_{5}+3{\text{H}}_{2}\text{O}+\text{HCl}\to {\text{Cu}}_{3}{\left({\text{AsS}}_{4}\right)}_{2}+2{\text{H}}_{3}{\text{AsO}}_{4}+5{\text{C}}_{2}{\text{H}}_{4}\text{O}+2\text{CuS}+\text{HCl}$$

The amount of arsenic(V) sulfide and copper(II) chloride taken for the reactions was 106.8 mg As_2_S_5_ and 110.45 mg CuCl_2_·2H_2_O for the 2nd reaction, and 145 mg As_2_S_5_ and 200 mg CuCl_2_·2H_2_O for the 4th reaction.

The reaction mixtures were stirred on a magnetic stirrer for 180 min. The pH of the reaction carried out in water medium was kept at 4–5, and the pH of the reaction carried out in ethylene glycol medium was kept at 5–6. After the process was completed, the sediments were filtered and washed with distilled water and ethanol. In order to homogenize the obtained samples, they were stored in a microwave oven for 48 h at a temperature of 333–353K by adding ultrapure water. After the process was completed, the obtained precipitates were filtered again, first washed with distilled water and then with ethanol, and dried under vacuum at 353 K.

## 3. Results and discussion

### 3.1. Effect of the pH of the solution

In the process, depending on the concentration of hydrogen ions, a precipitate of different composition can be formed. Therefore, the effect of the pH of the environment on the complete precipitation of the compound was studied. As can be seen from the reaction equations, hydrochloric and arsenic acids are formed in the solution during the process. Therefore, after adding copper(II) chloride to the solutions, the pH of the environment is within the range of 2–3. A 0.1 M ammonium hydroxide solution was used to increase the pH of the medium. It was determined that the formation of the thio compound is observed in the range of pH 2–5 for the water environment, and pH 2–6 for the ethylene glycol environment. At pH < 2 and pH > 7, the decomposition of the compound occurs. When the concentration of H^+^ ions in the solution is within the normal limit, compounds are decomposed according to the following reaction equations:


(5) $${\text{Cu}}_{3}{\left({\text{AsS}}_{4}\right)}_{2}+6{\text{H}}^{+}\to 3{\text{Cu}}^{2+}{\text{As}}_{2}{\text{S}}_{5}↓+3{\text{H}}_{2}\text{S}↑$$

The volume of the reaction products obtained during the decomposition process was determined by gravimetric methods^3^. Free sulfur formed by the reduction of hydrogen sulfide with iron (III) chloride was determined by the gravimetric method^4^. It was determined that the decomposition process accelerates when the temperature increases (T > 363 K).

When pH > 7, the concentration of OH^−^ ions in the solution increases. Due to this, oxythioduses are formed in the solution and are difficult to separate from the solution:


(6) $${\text{Cu}}_{3}{\left({\text{AsS}}_{4}\right)}_{2}+3{\text{OH}}^{-}\to {\text{Cu}}_{3}{\left(\text{OH}\right)}_{3}{\text{AsS}}_{4}+{{\text{AsS}}_{4}}^{3-}$$

From the conducted researches, it was determined that the optimal conditions for obtaining the compound under the reaction equations are pH 2–5 for the water environment, and pH 2–6 for the ethylene glycol environment. Furthermore, the temperature for both environments was determined to be 323–353 K and the time 150–180 min. Considering that the solubility of arsenic(V) sulfide in water is 0.0014 g/L, it can be said that no arsenic should enter the filtrate. However, during the course of the reaction, enough arsenic passed into the filtrate. From the results of chemical analysis, it was found that 3/8 of arsenic in the reaction carried out in the water medium, and 1/2 of the arsenic in the reaction carried out in the ethylene glycol medium passes to the filtrate^5^. As a result, the amount of arsenic taken, the amount of the obtained compounds and the amount of arsenic passing into the filtrate correspond to each other, indicating that the process is taking place in the direction of obtaining copper(II) thioarsenate. The impact of the concentration of hydrogen ions on the formation of the considered Cu_3_(AsS_4_)_2_ compound was studied, and the results are given in Tables 1 and 2.

**Figure 1:**
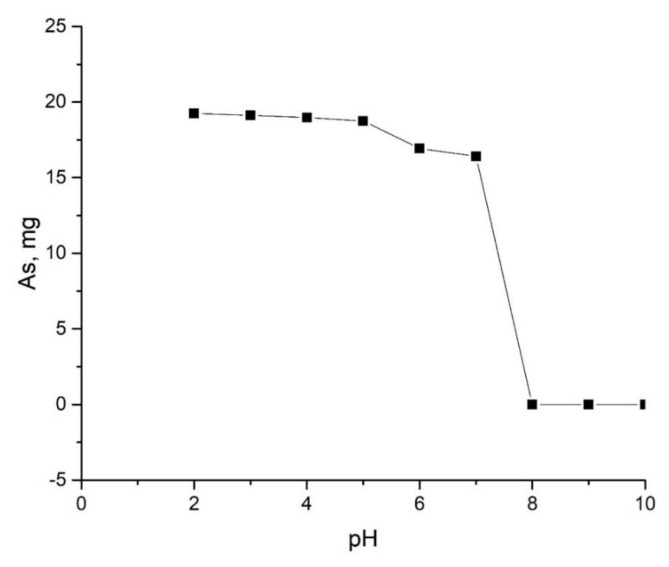
Graph of the dependence of the amount of arsenic passed into the solution on the pH of the environment during the experiment carried out in the water environment.

**Figure 2:**
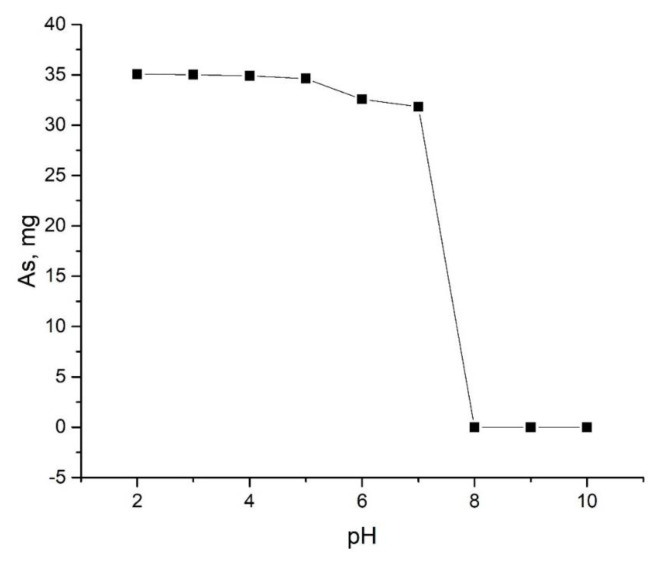
Graph of dependence of the amount of arsenic passed to the solution on the pH of the medium during the experiment carried out in ethylene glycol medium.

As can be seen from Tables 1 and 2, Figures 1 and 2, when the concentration of hydrogen ions of the solution increases, the amount of copper in the solution decreases. Complete consumption of copper ends at pH = 2–4 and pH = 2–5 for samples in water and ethylene glycol solutions, respectively. At this time, the theoretical amount of arsenic partially passes into the solution. Graphs of dependence of the amount of arsenic passing into the solution on the pH of the environment were constructed (Figures 1, 2). In general, as can be seen from the graphs, the amount of arsenic that passes into the solution remains relatively unchanged in the range of pH = 2–5. Therefore, it can be concluded that the experiments carried out in the range of pH 2–5 went in the direction of obtaining the copper(II) thioarsenate compound intended for reactions 2 and 4.

### 3.2. TG/DTG/DTA analysis

**Figure 3:**
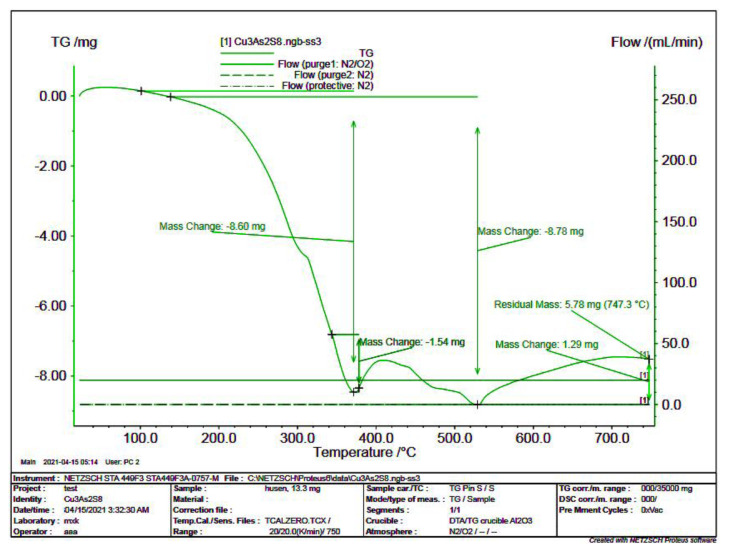
TG analysis curve of the thio compound obtained in a water environment.

In order to specify the stoichiometric composition of the synthesized thio compound in the aqueous environment, TG analysis was performed in the derivatograph (Figure 3). As can be seen from the thermogram, the 13.3 mg sample was heated to a temperature of 1023 K. Theoretically, the sample is known to contain 5.69 mg of sulfur and 3.33 mg of arsenic. Mass losses during the analysis were 5.0 mg at 573 K, 3.78 mg at 798 K, and a total loss at that temperature of 8.78 mg. This includes sulfur and arsenic. The residue of 5.78 mg at the temperature of 1023 K corresponds to the amount of copper(II) oxide. These losses occurred due to oxidation and sublimation of sulfur and arsenic in the sample. According to the TG analysis results, it was determined that the simple formula of the compound obtained in the water environment corresponds to Cu_3_(AsS_4_)_2_.

**Figure 4:**
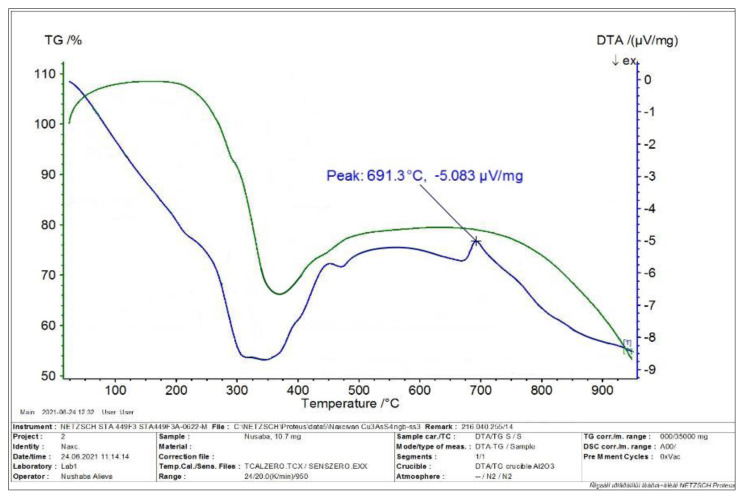
DTA curve of the sample obtained in a water environment.

From the DTA curve in Figure 4, it is clear that the sample obtained in water environment undergoes polymorphic transformation in the temperature range of 573–623 K and melts at the temperature of 964.3 K. Contrary to TG analysis, the melting temperature of the synthesized thiocompound corresponded to the melting temperature of the copper(I) thioarsenate compound.

**Figure 5:**
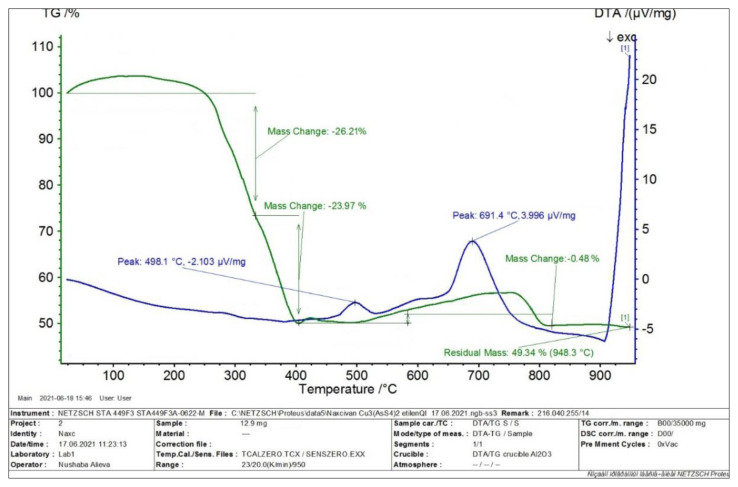
TG and DTA thermogram of the sample obtained in ethylene glycol medium.

In order to determine the stoichiometric composition of the synthesized thio compound in ethylene glycol medium, TG and DTA analysis were performed in the derivatograph, and the graph of the analysis result is given in Figure 5. The sample obtained in ethylene glycol medium was heated to 1223 K. It is clear from Figure 5 that there is a 50.66% loss from a 12.9 mg sample taken based on TG analysis. According to the DTA analysis, endothermic effects are observed at 2 points in Figure 5, which are at temperatures of 771.1 K and 964.4 K, respectively. The recording of the melting point at 964.4 K in the DTA curve and the amount of weight loss of the sample in the TG curve suggest that the stoichiometric composition of the obtained sample corresponds to the Cu_3_AsS_4_ compound.

### 3.3. XRD analysis

**Figure 6:**
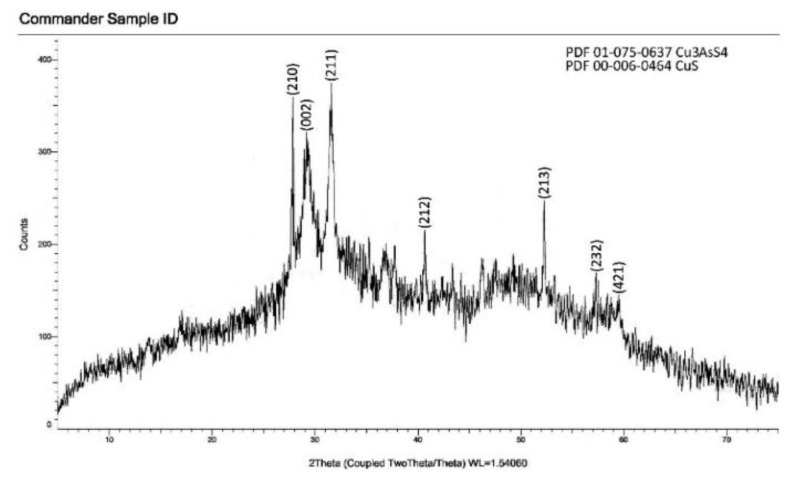
Diffractogram of Cu_3_AsS_4_ compound obtained in water environment.

**Figure 7:**
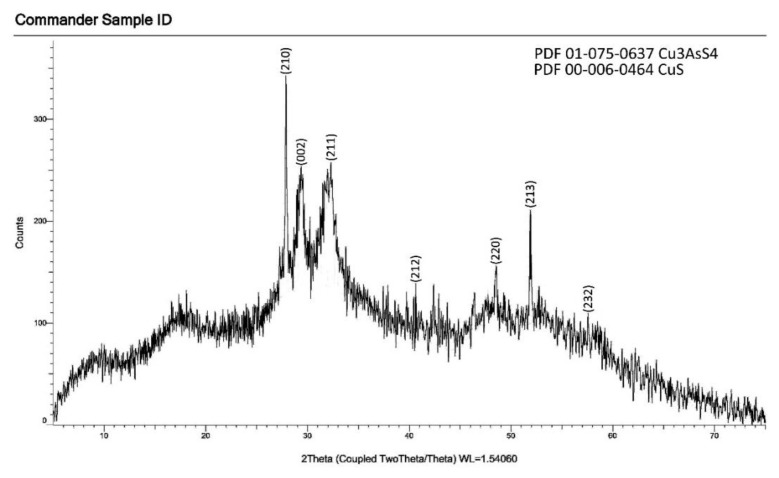
Diffractogram of Cu_3_AsS_4_ compound obtained in ethylene glycol medium.

The composition of samples obtained in water and ethylene glycol environments was investigated by the RFA method, and the results are shown in Figures 6 and 7. The obtained samples were thermally processed for 2 h at a temperature of 528 K in a vacuum (~10^−2^ Pa), and their composition was checked by RFA. It was found that the composition of the sample consists of copper(I) thioarsenate compound. The values of the intensity maxima in the diffractogram (Cu_3_AsS_4_–PDF 01-075-0637, CuS –PDF 00-006-0464) corresponded to the standards. Thus, based on RFA results, it was confirmed that copper(I) thioarsenate is formed from the interaction of arsenic(V) sulfide with copper(II) chloride in the water environment.

### 3.4. SEM imaging

**Figure 8:**
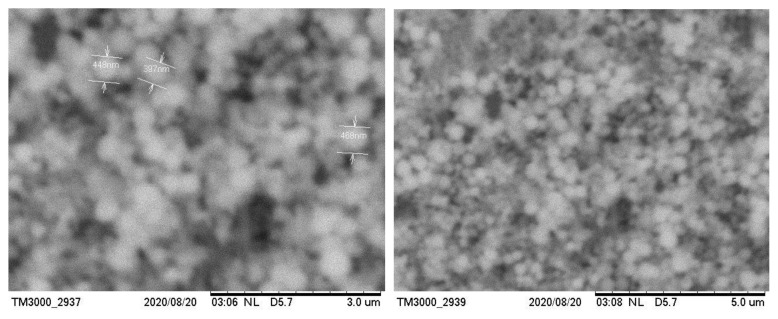
SEM images of Cu_3_AsS_4_ compound obtained in aqueous environment.

**Figure 9:**
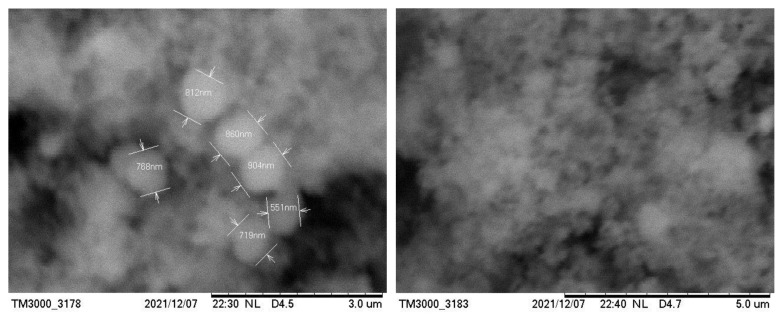
SEM images of Cu_3_AsS_4_ compound obtained in ethylene glycol medium.

The micromorphology and particle size of the obtained copper(I) thioarsenate compounds were studied using a SEM device (Figures 8, 9). Adhesion was observed between the particles of copper(I) thioarsenate compound deposited in the water environment, and it was determined that it consists of microparticles. As can be seen from the SEM images, the size of the particles in the 3 and 5 μm field is composed of aggregates of spherical particles in the range of 400–600 nm. At the same time, SEM images of the sample obtained in ethylene glycol medium were taken in the area of 3–5 μm, and it was observed that the particles are tightly connected to each other in the form of cotton.

### 3.5. Elemental analysis

**Figure 10:**
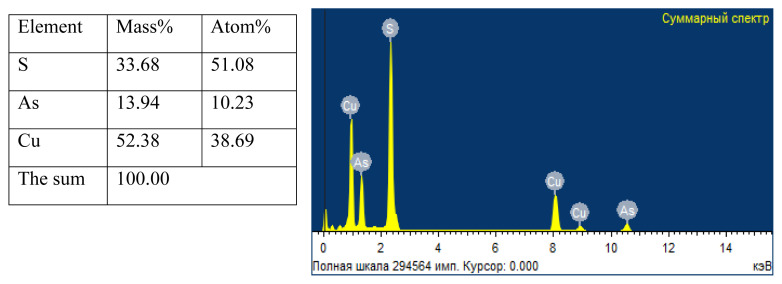
Element composition and energy-dispersive spectrum of Cu_3_AsS_4_ compound obtained in water environment.

**Figure 11:**
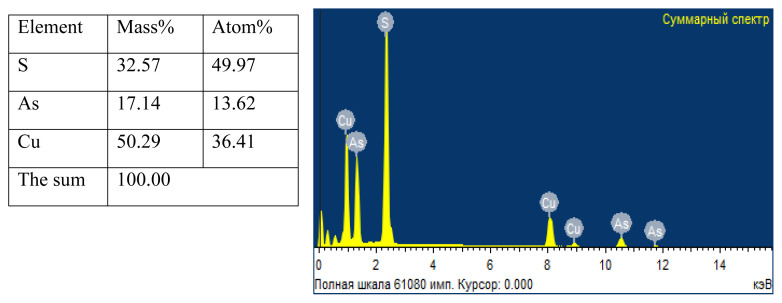
Elemental composition and energy-dispersive spectrum of Cu_3_AsS_4_ compound in ethylene glycol medium.

The percentage composition of the elements in the copper(I) thioarsenate samples obtained in water and ethylene glycol media and the spectroscopic data of the compound, and the energy-dispersive spectrum of the compound was drawn (Figures 10, 11). According to the obtained results, the mass and atomic ratios of copper, arsenic, sulfur in the sample taken in the water environment are 52.38%, 13.94%, 33.68% and 38.69%, 10.23%, 51.08%, respectively, while mass and atomic ratios of the copper, arsenic, sulfur in the sample taken in the ethylene glycol environment are determined to be 50.29%, 17.14%, 32.57% and 36.41%, 13.62%, 49.97%, respectively. Based on the results of elemental analysis, it was determined that the simple formula of compounds obtained in ethylene glycol and water environment corresponds to Cu_3_AsS_4_.

## 4. Conclusion

According to the 2nd and 4th reactions, the research work was performed in water and ethylene glycol environments. A number of analysis methods (TG, DTA, X-ray, elemental, and SEM) were used to determine the composition of the samples. The optimal conditions for the reactions carried out in both environments were determined. It was determined that the formation of the thio compound is observed in the range of pH 2–5 for the water environment, and pH 2–6 for the ethylene glycol environment. At pH < 2 and pH > 7, the decomposition of the compound occurs. The obtained results suggest that, contrary to the 2nd and 4th reactions, the composition of the obtained samples consisted of Cu_3_AsS_4_. Thus, the amount of losses in the samples during the TG analysis, the presence of endothermic effects in the curves of the DTA analysis graphs at temperatures of 964.4 K indicated the acquisition of the Cu_3_AsS_4_ compound in the conducted experiments. In addition, X-ray diffraction analysis confirmed that the composition of the sample mainly consists of Cu_3_AsS_4_ compounds in the intensity maxima in the diffractograms. From the SEM analysis of the obtained samples, it was clear that the particles in the 3 and 5 μm field of the compound obtained in the water medium were spherical (particles in the range of 400–600 nm), while the particles of the compound obtained in the ethylene glycol medium consisted of cotton-like particles closely connected to each other. Finally, the elemental analysis results of the mass and atomic ratios of copper, arsenic, sulfur in the sample taken in the water environment are 52.38%, 13.94%, 33.68% and 38.69%, 10.23%, 51.08%, respectively, while mass and atomic ratios of the copper, arsenic, sulfur in the sample taken in the ethylene glycol environment are determined to be 50.29%, 17.14%, 32.57% and 36.41%, 13.62%, 49.97%, respectively. It is confirmed that the obtained compound is compatible with Cu_3_AsS_4_ stoichiometry.

## Figures and Tables

**Table 1:** Dependence of sample formation in a water environment on the concentration of hydrogen ions.

The amount of As_2_S_5_ taken up, mg	The pH of the solution	The amount of CuCl_2_·2H_2_O, mg	The amount of precipitate obtained, mg		The amount of arsenic that passes into the solution, mg	
			Theoretical	Experimental	Theoretical	Experimental
106.80	10	110.45	128.76	-	19.37	-
-	9	-	-	-	-	-
-	8	-	-	-	-	-
-	7	-	-	110.74	-	16.41
-	6	-	-	115.36	-	16.93
-	5	-	-	126.47	-	18.74
-	4	-	-	127.89	-	18.97
-	3	-	-	128.06	-	19.12
-	2	-	-	128.55	-	19.25

Tem-r: 323K, Time-180 min.

**Table 2:** Dependence of sample formation in ethylene glycol medium on concentration of hydrogen ions.

The amount of As_2_S_5_ taken up, mg	The pH of the solution	The amount of CuCl_2_·2H_2_O, mg	The amount of precipitate obtained, mg		The amount of arsenic that passes into the solution, mg	
			Theoretical	Experimental	Theoretical	Experimental
145.00	10	200.00	185.15	-	35.15	-
-	9	-	-	-	-	-
-	8	-	-	-	-	-
-	7	-	-	167.03	-	31.84
-	6	-	-	176.21	-	32.56
-	5	-	-	182.73	-	34.63
-	4	-	-	183.45	-	34.89
-	3	-	-	184.81	-	35.01
-	2	-	-	184.97	-	35.07

Tem-r: 323K, Time-180 min.

## Footnotes

1 Tesfaye F, Taskinen P. (2010). Thernmodynamics and Phase Equilibria in the (Ni, Cu, Zn)-(As, Sb, Bi)-S Systems at Elevated Temperature (300–900 °C) [online]. Website https://urn.fi/URN:NBN:fi:aalto-201410132803 [accessed 4 April 2023].

2 Studenyak IP, Molnar ZR, Makauz II, Pop MM, Daróci L et al. (2018). Deposition and optical absorption studies of Cu–As–S thin films. Semiconductor physics, quantum electronics & optoelectronics [online]. Website http://nbuv.gov.ua/UJRN/MSMW_2018_21_2_11 [accessed 13 April 2023].

3 Korostelev P.P. (1985). Titrimetric and gravimetric analysis in metallurgy: Handbook. Metallurgy, Moscow: Moscow Metallurgy Publishing House [online]. Website http://www.chemnet.ru/rus/books/korostelev/welcome.html [accessed 14 January 2020].

4 Charlot G. (1965). Methods of analytical chemistry. Quantitative analysis of inorganic compounds. Moscow: Chemistry [online]. Website http://libarch.nmu.org.ua/handle/GenofondUA/78547 [accessed 17 January 2020].

5 Nemodruk A.A. (1976). Analytical Chemistry of Arsenic. Moscow: Nauka Publishing House [online]. Website https://libarch.nmu.org.ua/handle/GenofondUA/58964 [accessed 22 January 2020].
